# In Silico and In Vitro Studies of Antibacterial Activity of Cow Urine Distillate (CUD)

**DOI:** 10.1155/2024/1904763

**Published:** 2024-01-08

**Authors:** LokRaj Pant, Shankar Thapa, Bibek Dahal, Ravindra Khadka, Mahalakshmi Suresha Biradar

**Affiliations:** ^1^Department of Pharmacy, Sunsari Technical College, Dharan, Nepal; ^2^Department of Pharmacy, Universal College of Medical Sciences, Bhairahawa, Nepal; ^3^Department of Pharmacy, Madan Bhandari Academy of Health Sciences, Hetauda, Nepal; ^4^Department of Pharmaceutical Chemistry, Al-Ameen College of Pharmacy, Bengaluru 560027, Karnataka, India

## Abstract

Cow urine distillate (CUD) is a traditional Indian medicine used to treat various diseases, including bacterial infections. However, there is limited evidence to support its use as a medicine, and its safety and efficacy have not been thoroughly studied. In this study, we evaluated the antibacterial activity of CUD against five bacterial strains using in vitro and in silico approaches. In vitro experiments showed that CUD has significant antibacterial activity against all tested strains with a zone of inhibition (ZOI) ranging from 13 to 24 mm and minimum inhibitory concentration (MIC) values ranging from 12.5 to 50 *µ*g/ml. The results indicated that the 15% concentration of CUD displayed the highest antibacterial activity against *Staphylococcus aureus* and *Salmonella typhi*. To further investigate the antibacterial mechanism of CUD, we performed in silico docking studies of the active compounds of CUD with bacterial proteins involved in protein synthesis. Our results showed that 2-hydroxycinnamic acid (Δ*G* = −6.9 kcal/mol) and ferulic acid (Δ*G* = −6.8 kcal/mol) exhibited the best docking scores with the targeted proteins (DNA gyrase, PDBID: 4KFG). The hydrogen bonding interaction with amino acids Val71 and Asp73 was found to be crucial for their antibacterial activity.

## 1. Introduction

Cow urine has been used in traditional Indian medicine, known as Ayurveda, for thousands of years. Cow urine distillate (CUD) is a concentrate of cow urine that is used in Ayurvedic medicine as a treatment for various ailments. It is considered to have detoxifying and purifying effects and is used as a component in herbal formulations [1]. While some proponents of Ayurveda believe in its benefits, but it is not widely accepted or recognized as a medicine in modern and conventional therapy [2].

Antimicrobial agents, such as antibiotics and antiviral drugs, are critical for the treatment of infectious diseases. However, the overuse and misuse of these agents has led to the emergence of antibiotic-resistant bacteria, also known as “superbugs.” These superbugs are a major public health concern as they can cause serious infections that are difficult to treat, leading to prolonged illness and increased healthcare costs [3]. One of the major problems with traditional antimicrobial agents is that they are often synthetic compounds that can have toxic side effects and can also lead to the development of antibiotic-resistant strains of bacteria. In addition, many traditional antimicrobial agents are expensive and not accessible to everyone, especially in developing countries [4, 5].

CUD could be an alternative to traditional antimicrobial agents because it is a natural product that is readily available and inexpensive. There have been some studies on the antimicrobial and antioxidant properties of cow urine [6–8]. Traditionally (especially in Indian traditional medicine), it is strongly believed that CU can cure bacterial as well as viral diseases along with fever, anaemia, epilepsy, abdominal pain, constipation, and wound [9, 10]. This culture of folklore remedy is still in practice around the rural area of India and Nepal. In addition, CUD is believed to have anti-inflammatory and analgesic properties, and it may be useful in the treatment of certain types of cancer and diabetes [11, 12]. In South Asian country, CU is believed to cure cancer [13].

The molecular-level relationship between cow urine and bacterial proteins can provide the significant evidence of the bactericidal activity of CUD. However, more research is needed to understand the full potential of cow urine as an antimicrobial agent and to determine the safety and efficacy of using it for this purpose. The aim of the study is to investigate the antibacterial activity of CUD through both in vitro and in silico (molecular docking) methods in order to provide evidence-based results for its potential use as an antibacterial agent.

## 2. Materials and Methods

### 2.1. Ethical Approval

The ethical approval for the research was taken from the Internal Review Committee (IRC) at Sunsari Technical College (IRC No. ST15RE115).

### 2.2. Collection of Cow Urine and CUD Preparation

The milking cows were selected (10 cows of the same breed from the same farm) to collect the 20 ml of urine from each in a sterile container by randomized sampling technique. The sample was brought and stored in the refrigerator (4°C) until further use. A simple distillation process was used to collect the CUD at 100°C, and the distillate was stored in a sterile glass flask inside the refrigerator at 4°C [14]. After distillation, the residue was evaporated to obtain crude mass and submitted to prepare different required concentrations. The crude mass was taken, and 5%, 10%, and 15% concentrations with distilled water were prepared by the process called serial dilution.

### 2.3. Test Microorganism

The fresh pathogenic bacterial species were authenticated, collected, subcultured, and preserved. Bacterial strains consist of *Staphylococcus aureus* (Gram-positive), *Escherichia coli*, *Salmonella typhi*, and *Klebsiella species* (Gram-negative) [15].

### 2.4. Antibacterial Screening

Sterilization of Petri plates: the Petri dish was washed and sterilized in an autoclave at the temperature of 121°C for 15 minutes at 15 Ibs. Pressure by wrapping the Petri dish with aluminum foil.

#### 2.4.1. Preparation and Sterilization of Media and Nutrient Broth

About 19 grams of Muller–Hinton Agar (MHA) were placed in a 1000 ml conical flask, and 500 ml of distilled water was added from time to time. 1.3 grams of nutrient broth were placed in a 250 ml conical flask, and 100 ml of distilled water was added and both were heated to dissolve in a heating plate. After that cotton plug was placed in the mouth of the conical flask and covered with aluminum foil. It was put into an autoclave for sterilization at 121°C temperatures, at 15 Ibs pressure for 15 minutes [16].

#### 2.4.2. Subculture of Pure Bacterial Strain

When the media was cool, the nutrient broth was poured into four sterilized test tubes, and pure bacteria were added to the test tube with the help of a sterilized inoculating loop.

#### 2.4.3. Pouring of MHA in Petri Plates

When the media was cool, it was poured into sterilized Petri plates and allowed to solidify in an aseptic condition. After solidification, the Petri plates were wrapped with aluminum foil and stored in the refrigerator at 4°C.

#### 2.4.4. Preparation of Filter Paper Disc

The filter paper disc was prepared using Whatman No. 1 filter paper, and the paper disc was obtained with the help of the paper puncher to obtain a 6 mm diameter. The filter paper disc was placed in Petri plates and it was sterilized in a hot air oven at a temperature of 161°C for 2 hrs [17].

### 2.5. Assay of Antibacterial Activity

The antibacterial assay was carried out by the agar disc diffusion method. The antibacterial susceptibility testing was performed in three steps [18].

#### 2.5.1. Inoculation of Bacteria in the Media

In the aseptic condition under laminar airflow, the test organism was inoculated into the sterile Mueller–Hinton Agar by uniformly distributing it through the Petri plates with the help of a spreader. Then, proper labeling was done in each plate representing five regions, i.e., one for the positive control (ciprofloxacin 5 mcg), one for the negative control (dimethyl sulfoxide-DMSO), and three for different concentrations (5, 10, and 15%) of a sample.

#### 2.5.2. Incorporation of Samples into the Lawn Media

The prepared sterilized filter paper discs were impregnated with different concentrations of cow urine distillate and negative control DMSO separately. After that, the impregnated disc was placed in the inoculated agar plate in their region, and standard antibiotics (ciprofloxacin 5 mcg) were also placed, respectively.

#### 2.5.3. Incubation of the Plates

The impregnated plates with cow urine and standard antibiotics were incubated at 35°C for 24 hours. The diameter of the zone of inhibition indicates the antibacterial activity against test organisms. Each assay was carried out three times in this experiment, and the result obtained was recorded.

### 2.6. Minimum Inhibitory Concentration (MIC)

To determine the minimum inhibitory concentration (MIC), the broth dilution method was employed. Initially, 10 ml of Mueller–Hinton broth was added to sterilized test tubes and was then sterilized at 121°C for 15 minutes. A turbid solution was produced using the Mac-Farland turbidity standard scale. The test microorganism was introduced to sterilized test tubes containing 10 ml of normal saline and then incubated for 6 hours at 37°C. This was followed by diluting the test microorganisms until the turbidity matched that of the Mac-Farland scale, resulting in a concentration of approximately 1.5 × 10^8^ cfu/ml. The compound was then serially diluted in sterilized broth to achieve concentrations of 200, 100, 50, 25, and 12.5 *μ*g/ml, respectively. The MIC is the lowest concentration of the compound that resulted in no turbidity in the test tube after the broths had been incubated at 37°C for 24 hours [19–21].

### 2.7. Molecular Docking Process

The 3-dimensional (3D) structure of organic compounds and metabolic compounds present in the fresh cow urine were studied from different articles and downloaded from the PubChem database in “sdf” file format. The torsion angle and geometry minimization/energy minimization (MMFF94) were done by using MGL tool. The “sdf” file is converted into “pdbqt” from open-babel software [22, 23].

**Figure 1:**
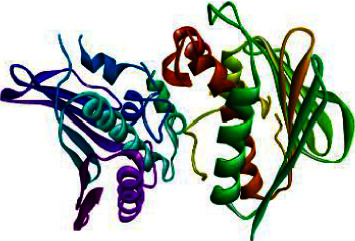
3D structure of DNA gyrase protein (PDBID: 4KFG).

The crystallographic structure of DNA gyrase B ATP binding domain of *Escherichia coli* protein (PDBID: 4KFG, resolution: 1.60 Å) [24] was retrieved from the Protein Data Bank (PDB) in “pdb” file format (Figure 1). The choice to use DNA gyrase (PDBID: 4KFG) for molecular docking investigations is driven by its essential function in bacterial DNA processing, which makes it an attractive candidate for antibiotic development. Utilizing the three-dimensional configuration of an enzyme assists in forecasting its interactions with individual chemical components of CUD, hence facilitating the identification of novel antibacterial drugs in the battle against antibiotic resistance [25]. Moreover, 4KFG has better resolution and conformation was validated with Ramachandran plot (Supplementary Material, Figure S1). The crystal structure of protein was purified by removing water molecule and co-crystal native ligand. The polar hydrogen was added to the protein structure in Discovery studio visualizer 2021 software [26, 27]. AutoDock Vina v.1.2.0 (https://vina.scripps.edu/) software was used for molecular docking studies [28, 29]. The grid dimension was set as default and blind docking was performed taking ciprofloxacin as reference. For the grid set up, the spacing was set as 1 Å and grid dimension was 50 dimensions for all *X*, *Y*, and *Z* axes. The center dimension was set as 14.299, 18.687, and −12.407 for the *X*-axis, *Y*-axis, and *Z*-axis, respectively. To validate the docking interaction, the redocking method was employed and the root mean square deviation (RMSD) value was calculated using PyMol 2.5.4 software (https://pymol.org/). The RMSD value less than 2 Å was considered as best fitted model [30, 31].

### 2.8. Main Component of CUD

The authentic research articles were downloaded from the Scopus-indexed journal by using “GC-MS (gas chromatography-mass spectroscopy) of cow urine” as prompt. Around 10 relevant articles were selected, and the main common chemical component from the article analysed [32–34]. We have chosen 7 common chemical components and submitted them for the docking studies.

## 3. Results

### 3.1. Organoleptic Characteristic

The physical appearance and the organoleptic characteristics of the CUD are analysed and reported (Table 1). The organoleptic characteristic provides evidence for compatibility and pleasing behavior for the conventional use of it. The organoleptic test was conducted as a preliminary analysis to identify the physical state of CUD.

### 3.2. Antibacterial Activity

**Figure 2:**
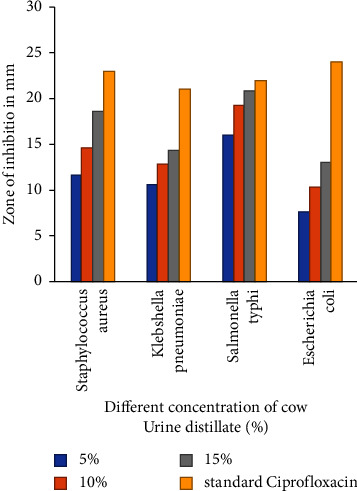
Zone of inhibition in diameter and concentration of CUD in %.

Antibacterial activity of CUD against four different strains, i.e., *Staphylococcus aureus* (Gram-positive), *Escherichia coli*, *Salmonella typhi*, and *Klebsiella species* (Gram-negative) is studied, DMSO is taken as negative control, and ciprofloxacin as a standard drug. The result of the study is shown in Table 2 and Figure 2.

### 3.3. Minimum Inhibitory Concentration

**Figure 3:**
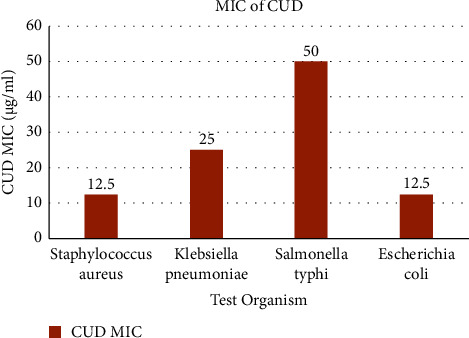
Minimum inhibitory concentration (MIC) of CUD.

Table 3 shows the MIC of CUD in different bacterial species. CUD is showing good activity on *Staphylococcus aureus* and *E. coli* (Figure 3). The minimum inhibitory concentration (MIC) of CUD was tested against different bacteria. The results show that CUD has antimicrobial activity against all the tested bacteria with MIC values ranging from 12.5 to 50 *μ*g/ml, which are higher than the reference compound.

### 3.4. Molecular Docking Result Analysis

**Figure 4:**
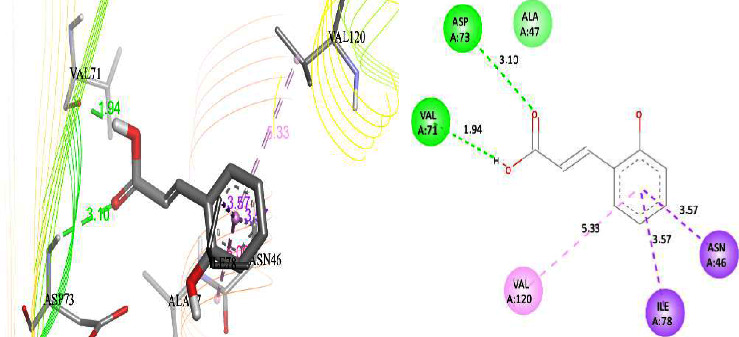
Interaction of compound 2-hydroxycinnamic acid (3D and 2D interactions).

**Figure 5:**
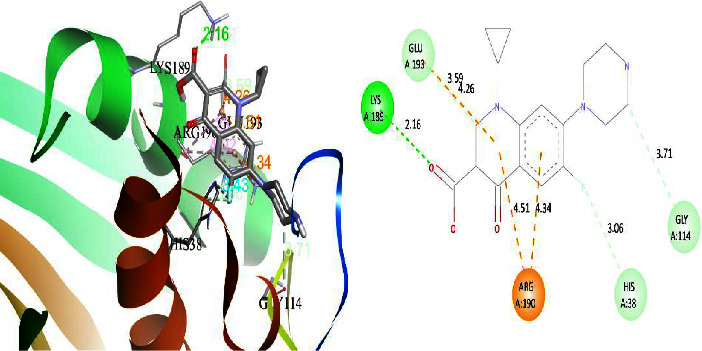
Interaction of reference ciprofloxacin with protein 4KFG (3D and 2D interactions).

**Figure 6:**
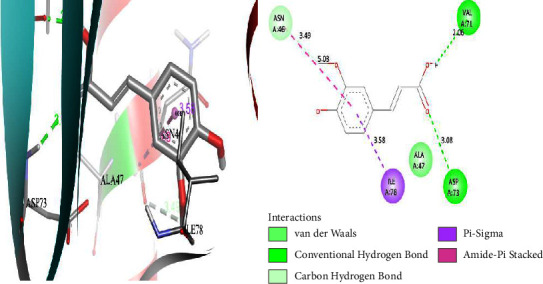
Interaction of compound ferulic acid with protein 4KFG (3D and 2D interactions).

The DNA gyrase enzyme is very essential in the cell transcription process of bacteria, and it controls the vital steps in the process. For docking study, we have taken the DNA gyrase B ATP binding domain protein of *Escherichia coli*. The protein (4KFG) has a native ligand (DOO) which has similar chemical fragment (aromatic and heterocyclic) as the cow urine chemical component (Supplementary Materials, Figure 2S). This resemblance makes 4KFG as perfect candidate as a target molecule for docking studies. The binding energy (in negative sign) and amino acid responsible for H-bonds were analysed and listed in (Table 4). From the result, it was observed that most of the ligand molecules have good binding interaction with protein. Among seven ligand molecules, 2-hydroxycinnamic acid showed the best binding energy of Δ*G* = 6.9 kcal/mol (Figure 4), with two conventional H-bond interactions. The amino acids Val71 and Asp73, with respective bond distances of 1.93 Å and 3.90 Å, were in-charge of H-bond interactions. The binding energy of 2-hydroxycinnamic acid was somewhat lower than reference ciprofloxacin (Δ*G* = 7.4 kcal/mol, Figure 5), but it had more hydrogen bond interactions than the reference. Similarly, ferulic acid (Figure 6) showed the binding energy of Δ*G* = −6.8 kcal/mol, which is slightly lower than the reference ciprofloxacin. The same amino acids (Val71 and Asp73) as 2-hydroxycinnamic acid participated in the interaction of ferulic acid with protein, although a variation in bond distance (2.06 Å and 3.08 Å) was detected. The highest conventional hydrogen bond interaction was demonstrated by gallic acid (Asp45, Glu42, and Ser108) with a binding energy of Δ*G* = −6.4 kcal/mol. Phenol displayed the lowest binding energy with only one hydrogen bond (Δ*G* = 5.2 kcal/mol). The calculated RMSD value of all the ligands molecules ranges 1.05 to 1.87 Å, which indicate the stability of ligands‐protein complex.

### 3.5. Main Component of CUD

Different spectral studies revealed the presence of a variety of aromatic and heterocyclic components in CUD which may help in the antibacterial activity. The prominent components of CUD include gallic acid, ferulic acid, cinnamic acid, allantoin, and 1-heneicosanol (Table 5).

## 4. Discussion

This study was conducted to analyse and evaluate the antibacterial activity of CUD by in vitro and in silico approach. The antibacterial activity of CUD against Gram-positive *Staphylococcus aureus* and Gram-negative *Escherichia coli*, *Salmonella typhi*, and *Klebsiella species* was evaluated at concentrations of 5%, 10%, and 15%. The results indicated that the 15% concentration of CUD displayed the highest antibacterial activity when compared to 5% and 10%. The greatest antibacterial activity was observed in *Salmonella typhi* and *Staphylococcus aureus* with diameters of 20.8 ± 0.6 mm and 18.6 ± 0.42 mm, respectively, at a 15% concentration, compared to the standard antibiotic ciprofloxacin. Our study shows different results compared to previous studies. Sathasivam et al. found smaller zones of inhibition for *Salmonella typhi* (10.4 ± 1.2 mm) [39], while Majhi and Bardvalli had similar results to our study [40]. Poornima et al. found that CUD had better antibacterial activity against Gram-positive bacteria, which is consistent with our findings [41]. In our study, the zone of inhibition for *E. coli* was 13 ± 0.8 mm at a 15% concentration of CUD, which is nearly 50% less than the standard drug ciprofloxacin (24 ± 1.0 mm in diameter). Jarald et al. found that crude cow urine had better antibacterial activity than CUD [42]. CUD may lose some of the potential components during the distillation process. This may hamper in potency of cow urine to inhibit the growth of bacteria. Moreover, Ahuja et al. reported similar findings to our study with a 14 mm zone of inhibition for *E. coli* [43]. These previous studies validate the findings of our research.

The MIC of CUD against *Staphylococcus aureus* and *E. coli* is 12.5 *μ*g/ml, while for *Klebsiella pneumoniae* and *Salmonella typhi* it is 25 and 50 *μ*g/ml, respectively. The MIC of the reference compound (ciprofloxacin) is 6.25 *μ*g/ml. These data suggest that CUD has antimicrobial activity against these bacteria, but its potency is lower compared to the reference compound. However, it is important to note that MIC values are just one measure of antimicrobial activity, and further studies are required to fully understand the effectiveness and safety of CUD as an antimicrobial agent.

Molecular docking is the process of interaction between the ligand and protein. It predicts the attachment of drug molecule to the binding site of receptors [44]. To explore the antibacterial mechanism of CUD, we have conducted in silico docking studies of CUD-active compounds with bacterial proteins responsible for protein synthesis. CUD has antibacterial activity, although the exact mechanism of action is not fully understood. Possible reasons for CUD's antibacterial activity include the presence of compounds such as urea, ammonia, osmolytes, and organic acids, which can denature proteins, disrupt cell membranes, cause dehydration, and have antimicrobial properties [45, 46].

Our findings revealed that 2-hydroxycinnamic acid (Δ*G* = −6.9 kcal/mol, Table 4) and ferulic acid (Δ*G* = −6.8 kcal/mol, Table 4) displayed the best docking scores with the targeted proteins, implying that CUD might function through this mechanism against the tested bacterial strains. The antibacterial activity of both the compounds was found to be associated with their hydrogen bonding interactions with amino acids, Val71 and Asp73.

According to reports, cow urine's antibacterial properties are attributed to the presence of 2-hydroxycinnamic acid, ferulic acid, gallic acid, cinnamic acid, phenol, carbolic acid, and allantoin. The peptides and derivatives in cow urine increase bacterial cell surface hydrophobicity, resulting in an impressive bactericidal effect. Cow urine is also known to boost the phagocytic activity of macrophages [47]. It was also claimed that cow urine has the ability to prevent the development of antibacterial resistance by blocking the R-factor, which is a component of the plasmid genome in bacteria [48].

Nautiyal and Dubey have found that CUD has antimicrobial activity against certain bacteria and fungi, and it is traditionally used as a disinfectant [35]. It is believed that cow urine contains a compound called urea which can denature bacterial proteins by breaking down their secondary and tertiary structures. This can disrupt the function of the protein and potentially inhibit the growth of the bacteria [49, 50]. Cow urine contains a compound called c-di-GMP (cyclic dimeric guanosine monophosphate), which is known to play a role in bacterial biofilm formation [51]. The study found that c-di-GMP present in cow urine can inhibit the production of a bacterial protein called SdiA, which is involved in biofilm formation. This suggests that cow urine may have the potential to inhibit the formation of bacterial biofilms [52]. CUD also contains osmolytes that can cause the dehydration and death of bacterial cells [53]. Moreover, the docking score of phenolic compounds (Δ*G* = −6.4 to −6.9 kcal/mol, Table 4) was found to be significant, indicating their potential role in the bactericidal activity of cow urine. Cinnamic and ferulic acids were also identified as important components that interacted strongly with DNA gyrase through hydrogen bonding. These results suggest a possible mechanism of action for cow urine. The high-level interaction between cow urine and DNA gyrase protein suggests it may be an effective solution to the problem of antibacterial resistance.

## 5. Limitation

Due to the resource's limitations, the research is lacking in the spectral analysis and isolation of individual components of CUD. The research does not claim that the individual components of the CUD contribute in the same way in vitro as computational studies have indicated. This will remain the verse of future scope.

## 6. Conclusion

In this study, it is found that CUD possesses significant antibacterial properties which support the claims of traditional practitioners. From the report of ZOI, around 15% of CUD significantly showed the antibacterial activity. It was also found that the 12.5 *μ*g/ml MIC value of CUD prominently inhibits the growth of bacteria. Molecular docking studies also clearly explain the molecular interaction of the CU constituent with the DNA gyrase protein. Ferulic acid and 2-hydroxycinnamic acid (constituents of CUD) showed the binding energy of 6.8 and 6.9 kcal/mol, respectively. However, an integrated approach is necessary to promote highly valuable virtues.

## Figures and Tables

**Table 1:** Organoleptic characteristic.

Organoleptic character	Fresh cow urine	Cow urine distillate
Colour	Golden yellow	Clear
Odor	Pungent	Strong pungent
Taste	Bitter	Slightly bitter

**Table 2:** Zone of inhibition in diameter and concentration of CUD in %.

SN	Bacteria	Concentrations			Standard
		5%	10%	15%	Ciprofloxacin (5 mcg/ml)
1	*Staphylococcus aureus*	11.6 ± 0.80	14.6 ± 0.32	18.6 ± 0.42	23 ± 0.92
2	*Klebsiella pneumoniae*	10.6 ± 0.81	12.8 ± 0.80	14.3 ± 0.32	21 ± 0.68
3	*Salmonella typhi*	16 ± 0.90	19.3 ± 0.81	20.8 ± 0.6	22 ± 0.87
4	*Escherichia coli*	7.6 ± 0.4	10.3 ± 0.92	13 ± 0.8	24 ± 1.0

**Table 3:** Minimum inhibitory concentration (MIC) of CUD.

Test organism	CUD MIC (*μ*g/ml)	Reference (ciprofloxacin) MIC (*μ*g/ml)
*Staphylococcus aureus*	12.5	6.25
*Klebsiella pneumoniae*	25	6.25
*Salmonella typhi*	50	6.25
*Escherichia coli*	12.5	6.25

**Table 4:** Binding energy and hydrogen bond interaction amino acids.

SN	Name	Structure	H-bond interactive amino acids	Binding energy (Kcal/mol)	RMSD value
1	Gallic acid	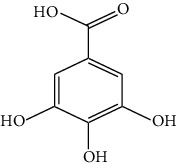	Asp45	−6.4	1.22
			Glu42		
			Ser108		

2	Ferulic acid	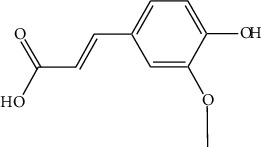	Val71	−6.8	1.60
			Asp73		

3	2-Hydroxycinnamic acid	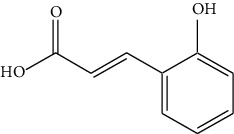	Val71	−6.9	1.05
			Asp73		

4	Cinnamic acid	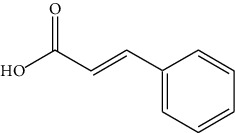	Val71	−6.7	1.15
			Asp73		

5	Salicylic acid	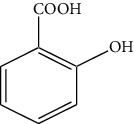	Asp73	−6.1	1.37
			Asn46		

6	Allantoin	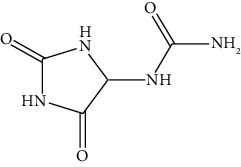	Asp73	−6.0	1.43
			Thr165		

7	Phenol	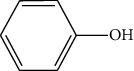	Val118	−5.2	1.87

8	Ciprofloxacin (reference)	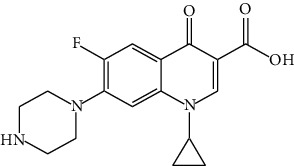	Lys189	−7.4	1.51

**Table 5:** GC-MS/FT-IR profiling of active component of CUD [35–38].

SN	Main component of CUD
1	1-Heneicosanol
2	Gallic acid
3	Ferulic acid
4	Pentadecanal
5	1-Hexadecanol
6	n-Heptadecanol-1
7	1,4-Dioxane-2,6-dione
8	2-Hydroxycinnamic acid
9	Cinnamic acid
10	Salicylic acid
11	Hexadecamethyl
12	Allantoin
13	Phenol
14	1-Triethylsilyloxyheptadecane

## Data Availability

The data used to support the findings of this study are available from the corresponding author upon request.

## Supplementary Materials

Supplementary Materials Figure 1S: Ramachandran plot of DNA gyrase (PDBID: 4KFG). Figure 2S: Native ligand (DOO) of DNA gyrase. IUPAC name: 6-fluoro-4-[(3aR, 6aR)-hexahydropyrrolo[3,4-b] pyrrol-5(1H)-yl]-N-methyl-2-[(2-methylpyrimidin-5-yl) oxy]-9H-pyrimido[4,5-b] indol-8-amine.
