# Supplementary material for: In Silico and In Vitro Studies of Antibacterial Activity of Cow Urine Distillate (CUD)
Source: Evid Based Complement Alternat Med. 2024 Jan 8;2024:1904763. doi: 10.1155/2024/1904763 (PMC10789515; doi:10.1155/2024/1904763)
Supplement: Supplementary Materials — Figure 1S: Ramachandran plot of DNA gyrase (PDBID: 4KFG). Figure 2S: Native ligand (DOO) of DNA gyrase. IUPAC name: 6-fluoro-4-[(3aR, 6aR)-hexahydropyrrolo[3,4-b] pyrrol-5(1H)-yl]-N-methyl-2-[(2-methylpyrimidin-5-yl) oxy]-9H-pyrimido[4,5-b] indol-8-amine. [file 1904763.f1.zip › Supplemetary material.docx]

*RESEARCH ARTICLE*

**In silico and in vitro studies of antibacterial activity of cow urine distillate (CUD)**

**LokRaj Pant^1^, Shankar Thapa^2*^,** **Bibek Dahal^1^, Ravindra Khadka^1^, Mahalakshmi Suresha Biradar^3^**

*^1^Department of Pharmacy, Sunsari Technical College, Dharan, Nepal*

*^2^Department of Pharmacy, Universal College of Medical Sciences, Bhairahawa, Nepal*

*^3^Department of Pharmaceutical Chemistry, Al-Ameen College of Pharmacy, Bengaluru-560027, Karnataka, India*

Correspondence should be addressed to Shankar Thapa; [tshankar551@gmail.com](mailto:tshankar551@gmail.com)

Supplementary materials:


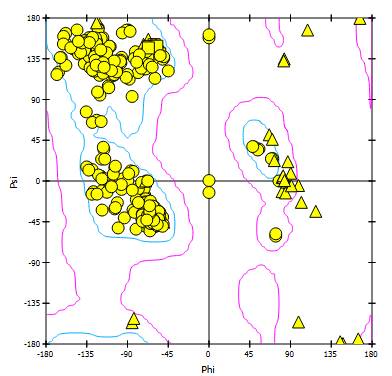


Figure 1S: Ramachandran plot of DNA gyrase (PDBID: 4KFG)


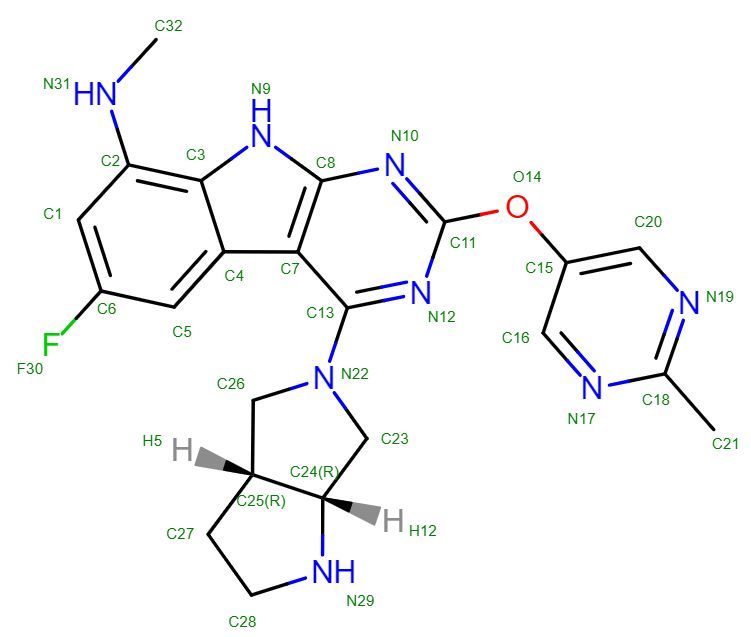


Figure 2S: Native ligand (DOO) of DNA gyrase. IUPAC name: 6-fluoro-4-[(3aR,6aR)-hexahydropyrrolo[3,4-b] pyrrol-5(1H)-yl]-N-methyl-2-[(2-methylpyrimidin-5-yl) oxy]-9H-pyrimido[4,5-b] indol-8-amine
